# WebGimm: An integrated web-based platform for cluster analysis, functional analysis, and interactive visualization of results

**DOI:** 10.1186/1751-0473-6-3

**Published:** 2011-01-17

**Authors:** Vineet K Joshi, Johannes M Freudenberg, Zhen Hu, Mario Medvedovic

**Affiliations:** 1 Laboratory for Statistical Genomics and Systems Biology, Department of Environmental Health, University of Cincinnati College of Medicine, 3223 Eden Av. ML 56, Cincinnati OH 45267-0056, USA

## Abstract

Cluster analysis methods have been extensively researched, but the adoption of new methods is often hindered by technical barriers in their implementation and use. WebGimm is a free cluster analysis web-service, and an open source general purpose clustering web-server infrastructure designed to facilitate easy deployment of integrated cluster analysis servers based on clustering and functional annotation algorithms implemented in R. Integrated functional analyses and interactive browsing of both, clustering structure and functional annotations provides a complete analytical environment for cluster analysis and interpretation of results. The Java Web Start client-based interface is modeled after the familiar cluster/treeview packages making its use intuitive to a wide array of biomedical researchers. For biomedical researchers, WebGimm provides an avenue to access state of the art clustering procedures. For Bioinformatics methods developers, WebGimm offers a convenient avenue to deploy their newly developed clustering methods. WebGimm server, software and manuals can be freely accessed at http://ClusterAnalysis.org/.

## Background

Identifying groups of co-expressed genes through cluster analysis has been successfully used to elucidate affected biological pathways and postulate transcriptional regulatory mechanisms. Methods for co-expression analysis of gene expression data have been extensively researched, and numerous clustering algorithms have been developed. New clustering algorithms often have been implemented as stand-alone computer programs, R packages, or both [1]. Numerous open source and commercial integrated analysis systems also implement multiple clustering algorithms. For example, MultiExperiment Viewer (MeV) [2] provides access to several clustering procedures as well as the mechanism for adding additional methods. The MeV+R package expands the utility of MeV to serve as a general "wrapper" and GUI for Bioconductor R packages [3]. Several web-servers for using specific clustering procedures exist where the web-interface is designed to gather data and necessary parameter values while the actual computation is performed on remote servers [4,5]. Separating the user interface from the computational infrastructure executing the algorithm, allows for computationally efficient implementations that utilize high-end HPC infrastructure to be leveraged against often computationally demanding clustering algorithms. Despite all these efforts, the methods most commonly used in practice are simple hierarchical clustering procedures implemented in Michael Eisen's cluster programs [6]. Results typically are visualized using the associated treeview program. "Interesting" clusters are selected by visual inspection, and functional enrichment analysis, if any, is performed using well-established online resources such as DAVID [7]. While seemingly ad-hoc, such general strategy has been remarkably successful in the analysis of genomics data.

The rationale for developing WebGimm is two-fold. First, sophisticated and better performing clustering methods are likely to be used more often if they are accessible through a streamlined and familiar interface requiring only minimal computational resources and no local installation. Second, an integrated web-based cluster/treeview-like platform that also incorporates functional enrichment analysis will further improve the utility of even simple hierarchical clustering procedures. We aimed to combine the "wrapper" model to facilitate access to clustering algorithms implemented in R, with the web-server model of deployment that obviates any local software installation.

To achieve these goals, we developed WebGimm, an open source general purpose clustering web-server infrastructure designed to facilitate the easy deployment of integrated cluster analysis servers based on clustering algorithms implemented in R. The design of our Java Web Start (JWS) client was modeled after the familiar *cluster/treeview *package. The version of the software deployed on our server implements multiple infinite mixture model based clustering procedures [1,8-11] as well as the most commonly used classical clustering procedures (hierarchical clustering and *k*-means clustering). In addition, functional analysis using the CLEAN framework and FTreeView browser [12] are integrated within the cluster analysis framework.

## Implementation

**Figure 1:**
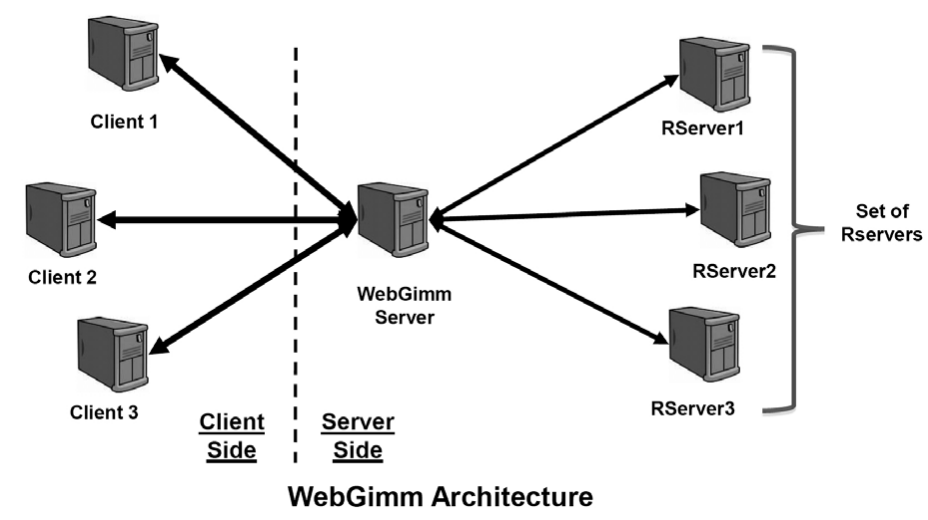
**WebGimm Architecture**. The complete system consists of three logical modules, the Client(s), the WebGimm server, and the R server(s).

WebGimm is an open source general purpose clustering web-server infrastructure designed to facilitate the easy deployment of integrated cluster analysis servers based on clustering algorithms implemented in R. The system consists of a Java GUI client deployed using the Java Web Start (JWS), and the server-side infrastructure designed around Java-based WebGimm server and multiple computing R servers. The server architecture is shown in Figure 1.

The design of the Java client is modeled after the familiar cluster/treeview package. The client's function is to pass user-specified analysis parameters and data to the server for analysis, and to facilitate viewing and downloading of analysis results. The server facilitates simple data centering and scaling, executing various clustering algorithms, performing functional enrichment analysis using CLEAN and viewing results of functionally annotated clustering results using Functional TreeView (FTreeView) [12]. The WebGimm server accepts data and computation requests from clients and assigns one of the R servers to perform the analysis using *Rserve *infrastructure http://www.rforge.net/Rserve/. R servers perform all computational tasks associated with cluster analysis and functional enrichment analysis by executing an R script with parameters supplied by the WebGimm server. R servers provide clients with feedback about the progress being made and also send a notification once the computation completes. Jobs are assigned to the servers in a round-robin fashion to evenly distribute the load among a "farm" of R servers.

## Results and Discussion

WebGimm serves as an integrated platform for cluster analysis, functional annotation of clustering results, and for exploring analytical results using the (FTreeView). The version of the software deployed on our server implements Gaussian Infinite Mixture Model (GIMM) based clustering procedures [1,8-10] as well as commonly used heuristic methods (hierarchical clustering and *k*-means clustering). In addition to providing a convenient tool for using GIMM, the integrated functional analysis and FTreeView browser provide a strong incentive to use the tool even when applying simple clustering procedures. The simplicity of deployment and the interface allows anybody with only conceptual understanding of cluster analysis to start using it with little effort.

**Figure 2:**
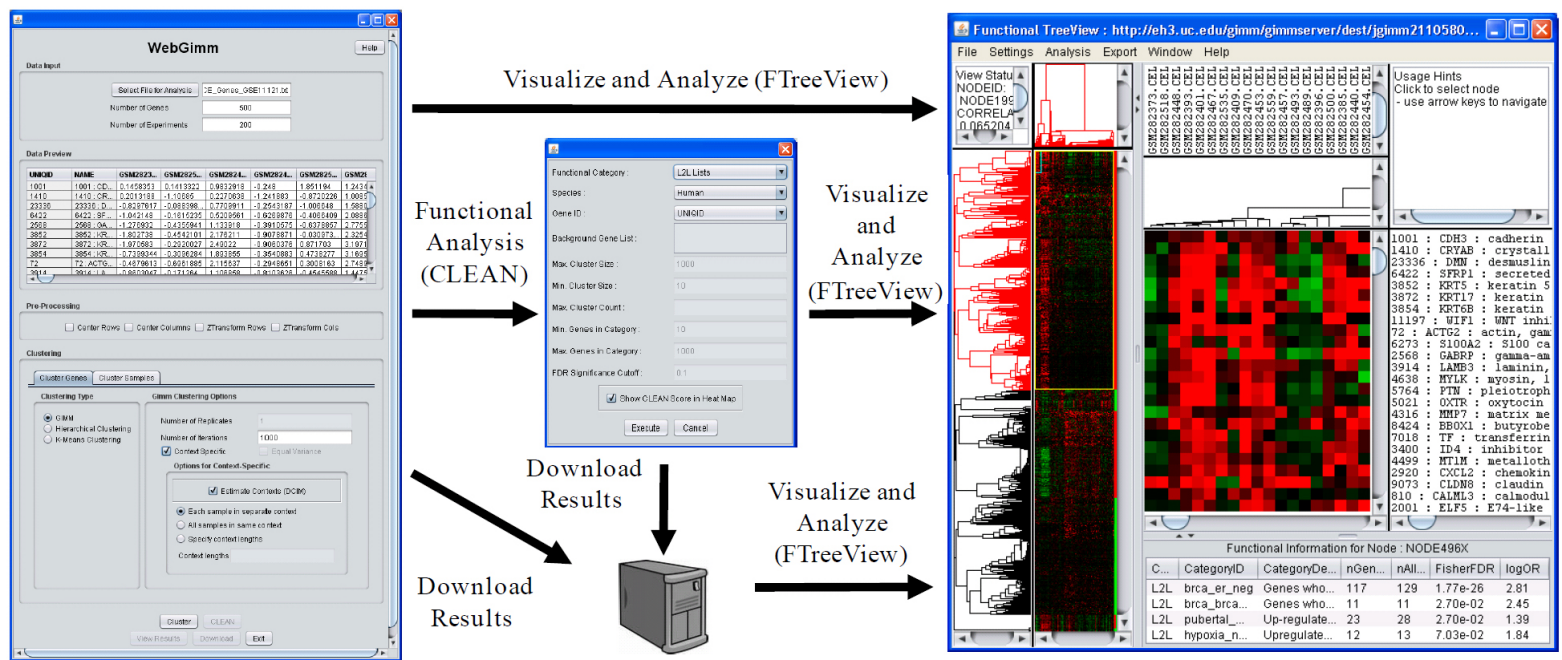
**Cluster analysis, functional enrichment analysis and integrated visualization of results using WebGimm platform**. The analysis flow is demonstrated by performing differential co-expression analysis using the DCIM algorithm [9]. The imported data is in the same format used by the Eisen's *cluster *program. After setting clustering paramers, the analysis is performed on the remote server. Results of the cluster analysis can be visualized using the integrated FTreeView browser, downloaded to the local computer, or further functional analysis can be performed. After the functional analysis, the results can again be visualized and analyzed using FTreeView.

Figure 2 demonstrates the use of the differential co-expression infinite mixture (DCIM) model [9] to cluster genes and group samples based on patterns of "differential co-expression", functionally annotate clustering results, and display them in FTreeView. After completion of the clustering analysis, the user has the option of examining the results using FTreeView, or performing functional enrichment analysis of the clustering results. In this case we used L2L lists [13] as the functional category to use in the CLEAN analysis and integrated analysis results are displayed in FTreeView.

The WebGimm infrastructure also provides a convenient way to implement and distribute newly developed clustering procedures. The complete code for client and server-side software, as well as instructions for deploying the server, can be downloaded from the support web site. By making simple modifications to the client GUI and the backend R scripts, Bioinformatics developers can deploy their own methods on their own servers in a way that is accessible to users without technical Bioinformatics expertise. Such deployment will likely increase the impact of their procedures, while allowing biomedical researchers to easily test state of the art analytical procedures and choose the one producing most meaningful results for their dataset at hand. Furthermore, separating the computational infrastructure from the user interface allows for a straightforward adoption of advanced computational paradigms. For example, the recent implementation of the hierarchical clustering using CUDA general purpose programming tools for NVIDIA Graphical Processing Units achieved 48-fold speed-up over typical desktop CPU using traditional sequential algorithm [14]. Implementing such algorithms on the computational server would not require any modifications of the WebGimm client.

## Availability and Requirement

Project name: WebGimm

Project home page: http://ClusterAnalysis.org

Operating system: platform independent client (tested on MS Windows, Mac OS and Linux), Linux-based web-server, platform-independent R packages

Programming language: Java, C++, MySQL, R

Other requirements: None

License: The tool is available online free of charge, and code is available based on GNU GPL.

Any restrictions to use by non-academics: None

## List of abbreviations used

CLEAN: Clustering Enrichment Analysis; DAVID: Database for Annotation, Visualization and Integrated Discovery; GIMM: Gaussian Infinite Mixture Model; JWS: Java Web Start.

## Competing interests

'The authors declare that they have no competing interests.

## Authors' contributions

VJ developed the complete server infrastructure and the JWS client. MM conceived and led the development of the software and the web-server. JF developed the server-side R scripts for performing cluster analysis and functional analysis. ZH develops and maintains the c++ GIMM code, VJ and MM wrote the paper. All authors read and approved the final manuscript.
